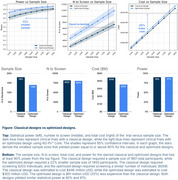# AI‐driven patient selection can drastically reduce sample sizes in Alzheimer's disease clinical trials

**DOI:** 10.1002/alz70859_100413

**Published:** 2025-12-25

**Authors:** Angela Tam, César Laurent, Adrián Noriega de la Colina, Christian Dansereau

**Affiliations:** ^1^ Perceiv AI, Montreal, QC Canada

## Abstract

**Background:**

Alzheimer’s Disease (AD) trials require excessively large sample sizes to be sufficiently powered because patients have highly heterogeneous disease trajectories. We propose to leverage AD‐Px™ Core, a predictive model that forecasts AD progression, to optimize patient selection in AD trials. We demonstrate how this patient selection can reduce sample sizes without sacrificing power or screening time to enable more cost‐effective trials.

**Method:**

We simulated trials with the CDR‐SB as the primary outcome measure and a treatment duration of 18 months. We applied inclusion criteria from recent pivotal early AD trials to real data from an aggregated pool of data comprising over 5,300 patients and 40,000 patient‐visits to select eligible participants for the simulations. As in a classical trial design, eligible participants were randomized to one of two arms (placebo or experimental) in the simulations. We then repeated these simulations, this time leveraging AD‐Px™ Core to optimize patient selection (pre‐randomization): Patients deemed by the model AD‐Px™ Core as unlikely to experience cognitive or functional decline were not randomized. Finally, we compared the classical design against the optimized design on their respective sample sizes, power, numbers to screen, and total costs. For each design, we aimed to find an optimal sample size with a target power equal to or above 90% to detect a 30% treatment effect.

**Result:**

The simulations showed that optimized trials have better statistical power than classical trials. This boost in power enables the optimized design to have a 22% smaller sample size, while maintaining 90% power and screening an equivalent number of patients, compared to a classical design. The reduction in sample size substantially lowers the total cost of the trial by $91 million USD (Figure).

**Conclusion:**

An AI‐driven patient selection method can drastically reduce the sample size and cost of a trial by at least 20%, while maintaining statistical power, thereby accelerating therapeutic development by enabling more cost‐effective trials.